# Scrapie versus Chronic Wasting Disease in White-Tailed Deer

**DOI:** 10.3201/eid3008.240007

**Published:** 2024-08

**Authors:** Zoe J. Lambert, Jifeng Bian, Eric D. Cassmann, M. Heather West Greenlee, Justin J. Greenlee

**Affiliations:** Oak Ridge Institute for Science and Education, Oak Ridge, Tennessee, USA (Z.J. Lambert);; US Department of Agriculture, Ames, Iowa, USA (Z.J. Lambert, J. Bian, E.D. Cassmann, J.J. Greenlee);; Iowa State University, Ames (Z.J. Lambert, M.H. West Greenlee)

**Keywords:** chronic wasting disease, scrapie, prions and related diseases, white-tailed deer

## Abstract

White-tailed deer are susceptible to scrapie (WTD scrapie) after oronasal inoculation with the classical scrapie agent from sheep. Deer affected by WTD scrapie are difficult to differentiate from deer infected with chronic wasting disease (CWD). To assess the transmissibility of the WTD scrapie agent and tissue phenotypes when further passaged in white-tailed deer, we oronasally inoculated wild-type white-tailed deer with WTD scrapie agent. We found that WTD scrapie and CWD agents were generally similar, although some differences were noted. The greatest differences were seen in bioassays of cervidized mice that exhibited significantly longer survival periods when inoculated with WTD scrapie agent than those inoculated with CWD agent. Our findings establish that white-tailed deer are susceptible to WTD scrapie and that the presence of WTD scrapie agent in the lymphoreticular system suggests the handling of suspected cases should be consistent with current CWD guidelines because environmental shedding may occur.

Prion diseases, or transmissible spongiform encephalopathies, result from the misfolding of a host’s endogenous prion protein and the accumulation of the misfolded form of the prion protein (PrP^Sc^) (*1*). Accumulation of PrP^Sc^ is associated with neurodegeneration and spongiform lesions that invariably kill the host (*1*). Prion diseases affect mammals: scrapie in sheep, chronic wasting disease (CWD) in cervids, bovine spongiform encephalopathy in cattle, and Creutzfeldt-Jakob disease in humans (*2*). The hallmark of transmissible spongiform encephalopathies is that the misfolded protein itself, devoid of the nucleic acid that drives viruses and bacteria (*1*), is able to transmit prion disease between animals (*3*).

 CWD was first identified in mule deer and black-tailed deer in 1967 (*4*). CWD has spread in cervids across North America and has been detected internationally (*5*). Within a species, prion diseases can occur as strains. Phenotypic features that can be used to differentiate strains may include host susceptibility based on prion protein sequence, incubation periods, age at clinical onset, tissue tropism, histologic patterns of PrP^Sc^ accumulation, biochemical and biological properties of PrP^Sc^, and range of susceptible species (*6*–*9*). Strain properties can be further differentiated by using rodent models (*10*). Although species outside the cervid family are susceptible to CWD (*11*–*17*), there is no evidence that the disease has been transmitted to humans (*18*).

Speculation about the origin of CWD has often implicated the classical scrapie agent of sheep (*19*–*22*), which is effectively transmitted to white-tailed deer intracranially and oronasally (*23*–*25*). That experimental disease, hereafter referred to as WTD scrapie, is lymphotropic (*23*,*24*), which means it is associated with environmental contamination and horizontal transmission (*26*–*28*) and could enable spread of the WTD scrapie agent in the cervid population (*29*–*31*).

Our purpose with this study was to examine the potential for white-tailed deer to transmit the WTD scrapie agent to other deer via oronasal exposure and to compare the disease phenotype to that of the CWD agent. We discovered that although differences exist between the WTD scrapie agent and the CWD agent in white-tailed deer, the presence of lymphoid involvement suggests that environmental contamination is highly likely. As the geographic distribution and disease incidence of CWD in white-tailed deer increases, information about the potential role of scrapie in the burgeoning CWD epidemic could assist in mitigation efforts. Our animal experiment was approved by the National Animal Disease Center Institutional Animal Care and Use Committee.

## Materials and Methods

Our study population comprised 3 white-tailed deer that were homozygous for glutamine at codon 95 and glycine at codon 96 (QQ95/GG96) of the *PRNP* gene. To enable comparison of WTD scrapie with CWD by the assays used in this study, we used samples from a deer experimentally inoculated with the CWD agent (National Animal Disease Center [NADC] identification [ID] 1548); the deer was of the same genotype and inoculated by the same route as the 3 deer in our study population. 

We oronasally inoculated deer with 1 mL of a 10% wt/vol brainstem homogenate in phosphate-buffered saline (PBS) from a white-tailed deer (NADC ID 18, publication ID 9 [*23*]) in which classical scrapie had developed (isolate no. 13-7 ARQ/ARQ [*32*]) after oronasal inoculation. We conducted antemortem rectal biopsies 17 months after inoculation. Animal caretakers observed the deer daily and euthanized them when they exhibited clinical signs (e.g., weight loss, hair loss, excessive salivation, diarrhea, and progressive weakness). We performed necropsies on the euthanized deer and collected the following samples: whole brain, cerebrospinal fluid, brainstem, spinal cord (cervical, thoracic, and lumbar segments), dorsal root ganglia, eyes, turbinate, nerves (optic, trigeminal, sciatic), lymph nodes (retropharyngeal, prescapular, mesenteric, popliteal), thymus, thyroid gland, trachea, esophagus, foregut (rumen, reticulum, omasum, abomasum), jejunum, ileum, cecum, recto-anal mucosa–associated lymphoid tissue, kidney, adrenal gland, liver, urine, spleen, lung, skin, and muscles (tongue, masseter, heart, diaphragm, triceps brachii, biceps femoris, psoas major, bladder). We collected 2 sets of tissue samples, froze 1 set, and collected the other in 10% formalin and embedded it in paraffin wax. We stained or immunolabelled embedded samples for microscopy and immunohistochemistry. Frozen samples of brainstem and retropharyngeal lymph node underwent enzyme immunoassay. We performed Western blots on brainstem, retropharyngeal lymph node, and cerebrum samples and further evaluated brainstem samples by dot blot PrP^Sc^ conformational stability assay and mouse bioassay.

For genotyping of the white-tailed deer, we extracted DNA before conducting PCR. We combined the DNA samples with PCR mix (5× buffer, deoxynucleotide triphosphates, primer #1, primer #2, dimethyl sulfoxide, Herculase II Fusion DNA polymerase [https://www.agilent.com], and double-distilled water) in a thermal cycler (Applied Biosystems, https://www.thermofisher.com) as previously described (*33*). We modified the program slightly from those previously described: 95°C for 5 minutes, 40 cycles of 95°C for 20 seconds, 54°C for 20 seconds, 72°C for 1 minutes, followed by 72°C for 7 minutes, and held at 4°C until samples were removed. We purified the PCR products by using Amicon Ultra Filters (30 kDa) (Sigma Aldrich, https://www.sigmaaldrich.com) according to manufacturer instructions. We ran the samples on an agarose gel (1%) and DNA sequenced them.

The enzyme immunoassay kit that we used is commercially available (HerdChek, IDEXX Laboratories Inc., https://www.idexx.com), and we followed manufacturer instructions to screen for PrP^Sc^ in the cerebrum, brainstem, retina, and retropharyngeal lymph nodes. We determined the negative cutoff threshold by using the negative control provided in the kit. We considered values above the optical density threshold positive. We quantified the misfolded prion protein in brainstem samples by making 2-fold dilutions to compare relative prion loads for cervidized mouse bioassays (Table 1).

**Table 1:** Two-fold dilutions of brainstem inoculum in cervidized mice inoculated with WTD deer scrapie agent, passage 1 or 2, compared with WTD CWD agent*

Weight/ volume, %	WTD CWD	WTD scrapie agent	
		Passage 1	Passage 2
10	3.847	3.758	4
5	3.721	3.395	4
1	3.657	1.973	3.784
0.50	3.828	1.363	3.291
0.25	3.643	0.674	2.74
0.125	3.224	0.432	1.808
0.0625	2.354	0.244	1
0.031	1.345	0.138	0.439

*The optical density of brainstem samples after 2-fold dilutions to compare relative load of misfolded prion protein in the inoculum (1% wt/vol) of cervidized mice (Tg12). CWD, chronic wasting disease; WTD, white-tailed deer.

We processed frozen tissues as 20% wt/vol protein homogenates by using PBS for Western blotting in a Bead Mill 24 homogenizer (Fisher Scientific; https://www.fishersci.com). Samples were digested by proteinase K (PK; 1 mg/mL) (Invitrogen, https://www.thermofisher.com) for 1 hour at 37°C with agitation (500 rpm), followed by reaction neutralization with Pefabloc (100 mg/mL) (Roche Diagnostics GmbH, https://www.roche.de) and incubation for 20 minutes at room temperature. We completed immunodetection of the misfolded prion protein on the cerebrum (frontal cortex), cervical spinal cord, retina, and retropharyngeal lymph nodes. Because of lack of tissue availability of the brainstem at the obex, we used cervical spinal cord. We prepared samples with lithium dodecyl sulfate sample buffer and 2-mercaptoethanol before loading them onto commercial-grade 12% SDS-PAGE (sodium dodecyl sulfate–polyacrylamide gel electrophoresis) gel and running for 45 minutes at 200 V in MOPS SDS running buffer (Invitrogen) with NuPageTM Antioxidant (Invitrogen) in the center chamber. We then transferred the gel to a polyvinylidene difluoride membrane and blocked with 3% bovine serum albumin in Tris-buffered saline with 0.05% Tween 20. We then probed blots with mouse monoclonal antibodies against the prion protein: 6H4, SHA31, 12B2, and P4, all at 1:10,000 dilution (0.1 μg/mL). The C-terminal antibody 6H4 recognizes amino acids PrP-Ov 148-156/PrP-Bov 155-163 (Prionics, https://www.prionics.com), and the SHA31 C-terminal antibody recognizes amino acids 144–155 (Bertin Bioreagent, https://www.bertin-bioreagent.com) of the prion protein. On the N-terminal of the prion protein, antibody 12B2 targets amino acids PrP-Ov 89-107/PrP-Bov 97-115 (Wageningen Bioveterinary Research, https://www.wur.nl) and antibody P4 targets amino acids PrP-Ov 93-99 (R-Biopharm AG, https://r-biopharm.com). We used an antimouse biotinylated sheep secondary antibody at a 1:400 dilution (Cytiva, https://www.cytivalifesciences.com) and a conjugated streptavidin-horseradish peroxidase at a 1:10,000 dilution (Cytiva) for amplification and signal detection. We incubated primary antibodies overnight in 4°C and the subsequent antibodies for 1 hour each at room temperature. Visualization was achieved by using electrochemiluminescence (Thermo Fisher Scientific, https://www.thermofisher.com and an iBright 1500 (Invitrogen). We used PageRuler Plus prestained protein ladder (Thermo Fisher Scientific) to demark relative weights.

For immunohistochemistry, we used 4-micrometer paraffin-embedded tissue sections, stained the tissues with hematoxylin and eosin, and used an automated Ventana Discovery XT staining machine (Roche Diagnostics, https://diagnostics.roche.com) for microscopic analysis of misfolded prion protein staining. After deparaffinization and rehydration, we treated samples with 98% formic acid for 5 minutes and then performed antigen retrieval at 121°C for 20 minutes by using Diva Decloaker (Biocare Medical, https://biocare.net). We then probed tissue sections with the primary antibody F99/97 and took images with a Nikon Eclipse 55i microscope (https://www.nikonusa.com) by using Infinity Analyze software (Lumera, https://www.lumenera.com).

We completed bioassays in cervidized mice (Tg12 [*34*]) that received inoculum from the brainstem of deer 1 (WTD scrapie P2; n = 15). For comparison, we inoculated brainstem from first-passage WTD scrapie agent into cervidized mice and derived from the same deer used for inoculation in this study (WTD scrapie P1; n = 25). We inoculated another group of cervidized mice with brainstem from a white-tailed deer with CWD (WTD CWD, n = 9); the mice were anesthetized and intracranially inoculated with 20 μL of 1% wt/vol brainstem homogenate in PBS. After inoculation, we monitored the mice for clinical signs, then euthanized and necropsied them until study completion.

We conducted a conformational stability assay of the misfolded prion protein by using 96-well plates and 5–50 μg of tissue homogenates from white-tailed deer. We denatured tissue homogenates in 0–5.5 M guanidine hydrochloride G7294 (GdnHCl) (Sigma Aldrich, https://www.sigmaaldrich.com) at room temperature for 1 hour. We then filtered the samples on Amersham Protran nitrocellulose membrane (Cytiva) with a Bio-Dot Microfiltration apparatus (Bio-Rad Laboratories, https://www.bio-rad.com), followed by 2 PBS washes and air drying of the membrane for 1 hour. We then incubated them with PK (5 μg/mL) in cell lysis buffer (50 mM Tris-HCl, pH 8.0, 150 mM NaCl, 0.5% sodium deoxycholate, 0.5% Igepal CA-630 [https://www.sigmaaldrich.com]) for 1 hour at 37°C. We inactivated digestion with PK with 2 mM phenylmethylsulfonyl fluoride. Denaturation of the membrane took 10 minutes in 3 M guanidine thiocyanate in Tris-HCl (pH 7.8) at room temperature. After 4 PBS washes, we blocked membranes with 5% nonfat milk in in Tris-buffered saline with 0.05% TWEEN 20 for 1 hour, then probed at 4°C overnight with SHA31 (Bertin Technologies, https://www.bertin-technologies.fr) diluted 1∶5,000, followed by horseradish peroxidase–conjugated goat antimouse IgG secondary antibody. We used ECL Plus (Pierce ECL Plus Western Blotting Substrate [Thermo Fisher Scientific]) to develop the membranes, a ChemiDoc imager (Bio-Rad) to take the images, and AzureSpot Pro analysis software (Azure Biosystems, https://azurebiosystems.com) to complete the signal analysis. We completed analysis on 3 biological replicates. We normalized absolute densitometric values by defining the smallest mean of each sample as 0 and largest mean as 1. To produce denaturation curves, we plotted relative levels of the undenatured PrP^Sc^, referred to as F*app* (apparent fractional change of unfolded PrP^Sc^), as a function of GdnHCl concentration. We used a nonlinear least-square 4-parameter sigmoidal dose-response regression with the half maximal denaturation concentration, [GdnHCl]_1/2_, calculated by using Graphpad Prism software (https://www.graphpad.com). We used the Student *t*-test to assess the statistical significance of [GdnHCl]_1/2_.

## Results

Of the 3 wild-type (QQ95/GG96) white-tailed deer oronasally inoculated with brainstem homogenate from a deer that succumbed to no. 13-7 classical scrapie, all either exhibited clinical signs (excessive salivation, hair loss, and weight loss) and were euthanized or found dead 21–25.8 months after inoculation (Table 2). Enzyme immunoassays performed on central nervous and lymphoreticular system tissues (Table 2) indicated that all 3 deer were positive for PrP^Sc^ in the brainstem. Deer 1 (optical density [OD] 4.00) and 2 (OD 3.26) had relatively more PrP^Sc^, indicated by greater optical density than in deer 3 (OD 1.79) in the brainstem. Regardless, all white-tailed deer had 4.0 PrP^Sc^ in the retropharyngeal lymph nodes. Only deer 1 was positive in the cerebrum and retina (OD 4.00).

**Table 2:** Misfolded prion protein presence and accumulation in multiple tissues from 3 white-tailed deer with white-tailed deer scrapie*

Identification	Deer 1	Deer 2	Deer 3
Animal data			
Genotype (95/96)†	QQ/GG	QQ/GG	QQ/GG
Incubation period, mo after inoculation	25.6	21	25.8
EIA OD			
Brainstem	4	3.26	1.79
Cerebrum	4	Not detected	Not detected
Retina	4	Not detected	Not detected
Retropharyngeal lymph nodes	4	4	4
Spongiform lesion on brainstem	+	+	+
Immunohistochemistry			
Antemortem rectal biopsy (17 mpi)	+	Insufficient	+
Brainstem	+	+	+
Cerebrum	+	Not detected	Not detected
Retina	+	+	+
Retropharyngeal lymph nodes	+	+	+
Palatine tonsil	+	+	+

*EIA OD, enzyme immunoassay optical density. †Homozygous for glutamine at codon 95 and glycine at codon 96 (QQ95/GG96) of the *PRNP* gene**.**

**Figure 1:**
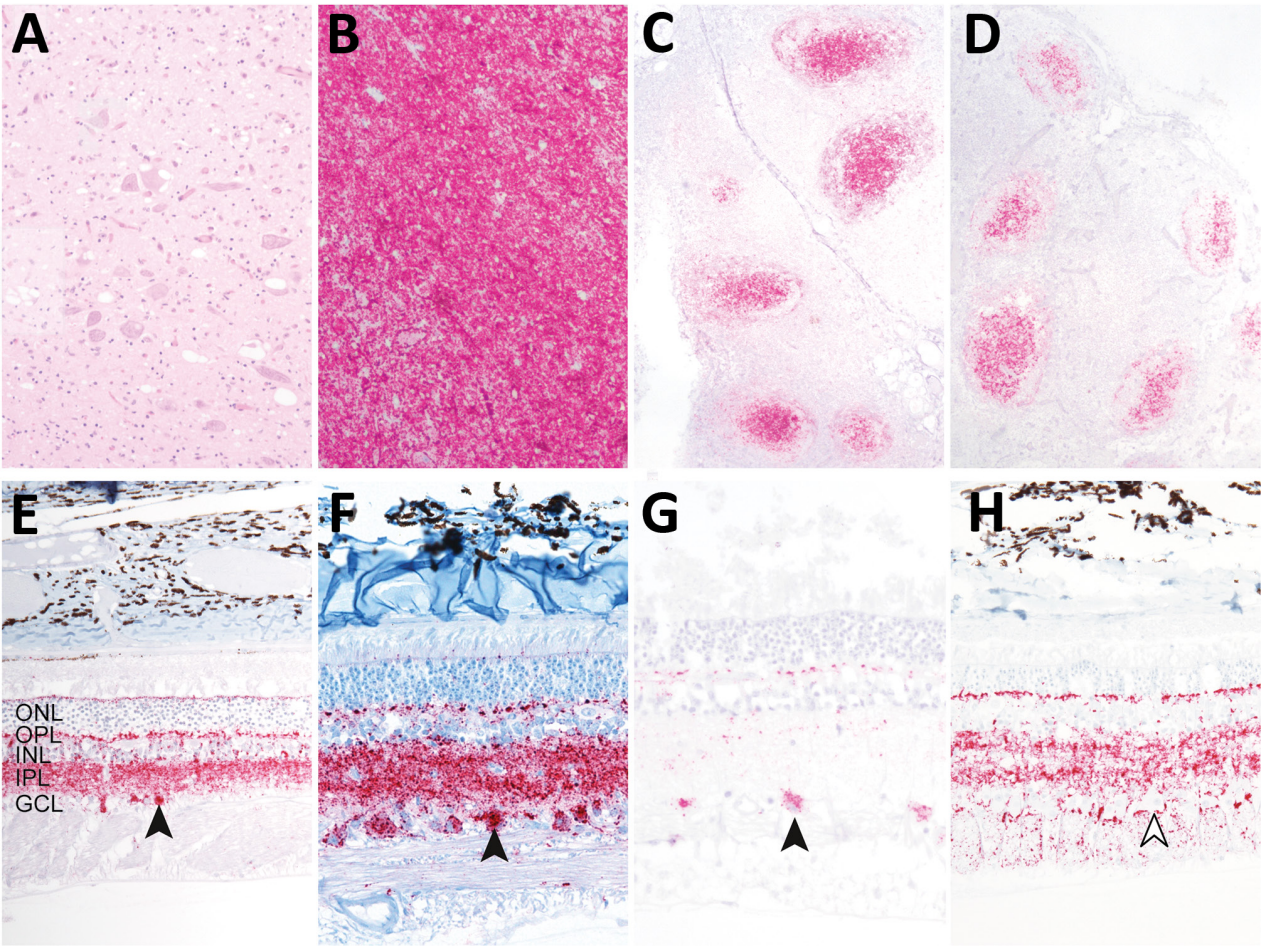
Immunohistochemistry demonstrating misfolded prion protein in white-tailed deer oronasally inoculated with white-tailed deer scrapie (WTD scrapie) agent in study of scrapie versus chronic wasting disease (CWD) in white-tailed deer. A) Vacuolation in the dorsal motor nucleus of the vagus in the brain stem at the level of the obex of each deer. B–D) Misfolded prion protein in the dorsal motor nucleus of the vagus in the brain stem at the level of the obex (B), palatine tonsil (C), and retropharyngeal lymph node (D) of each deer. E–H) Neurotropism of the scrapie form of the prion protein for retinal ganglion cells with scrapie agent (closed arrowheads) and not CWD (open arrowhead). E) Sheep scrapie retina; F) WTD scrapie, passage 1, retina; G) WTD scrapie, passage 2, retina; H) WTD CWD, retina. Hematoxylin and eosin staining; original magnification ×10 for panels A–D, ×20 for panels E–H. GCL, ganglion cell layer; INL, inner nuclear layer; IPL, inner plexiform layer; ONL, outer nuclear layer; OPL, outer plexiform layer.

Immunohistochemistry indicated spongiform lesions and misfolded prion protein accumulation in the dorsal motor nucleus of the vagus in the brainstem at the level of the obex in all 3 white-tailed deer inoculated with WTD scrapie (Figure 1, panels A, B). PrP^Sc^ accumulation was also detected in the palatine tonsils and retropharyngeal lymph nodes of each deer (Figure 1, panels C, D). Only deer 1 exhibited strong immunolabelling for misfolded prion protein in the retina (Figure 1, panel G). That deer also had the greatest level of spongiform lesions and misfolded prion protein accumulation in the dorsal motor nucleus of the vagus in the brainstem at the level of the obex. The PrP^Sc^ in the retina of deer 1 was abundant in the retinal ganglion cells (Figure 1, panel G), similar to that in the retinas of white-tailed deer (Figure 1, panel F) and sheep (Figure 1, panel E) inoculated with the no. 13-7 classical scrapie isolate from sheep. That finding differs from that of white-tailed deer with CWD, in which the retinal ganglion cells generally lack that type of accumulation (Figure 1, panel H). Deer 2 and 3 exhibited minimal immunolabelling for PrP^Sc^ in the retina (Table 2). Because staining was limited to the optic disk and plexiform layers in those deer, evaluation of retinal ganglion cells for PrP^Sc^ could not be completed.

**Figure 2:**
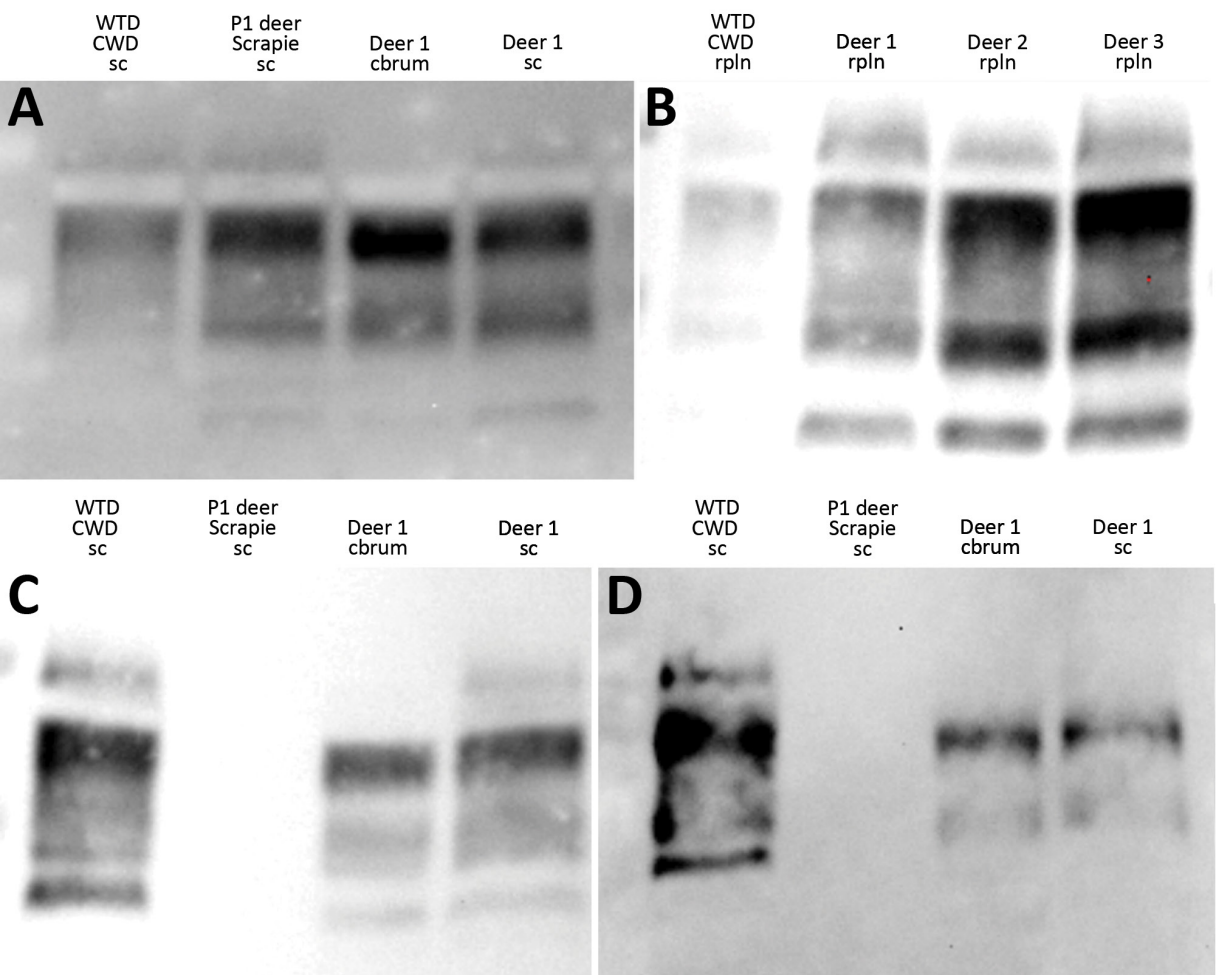
Epitope mapping on Western blots with antibodies that progress toward the N-terminal of the prion protein in study of scrapie versus CWD in white-tailed deer. C-terminal antibodies 6H4 (A) and SHA31 (B) were used to probe brain and lymphoid tissue (representative samples). C) Material from the second passage of WTD scrapie and CWD, both responsive to probing by the N-terminal antibody 12B2. D) WTD scrapie material showing no or low affinity to the N-terminal antibody P4. Cbrum, cerebrum; CWD, chronic wasting disease; P1, first passage; rpln, retropharyngeal lymph node; sc, cervical spinal cord; WTD, white-tailed deer.

Molecular profile differences were reported for tissues from white-tailed deer with first-passage WTD scrapie because brainstem was CWD-like (relatively higher kDa) and cerebrum was scrapie-like (relatively lower kDa) (*23*). Western blots were performed to evaluate whether the molecular profile differences would persist. Epitope mapping using different antibodies enabled assessment of approximate PK cleavage sites. When we used C-terminal antibodies (6H4 or SHA31), the molecular profile of the tissues from second-passage WTD scrapie was similar to that of the inoculum as well as tissues from white-tailed deer with CWD (Figure 2, panels A, B). When we used N-terminal antibody 12B2, the inoculum was nonreactive, but CWD and second-passage WTD scrapie isolates appeared similarly reactive (Figure 2, panel C). However, antibody P4 recognized an epitope further toward the N-terminal than 12B2, enough to distinguish between CWD and second-passage WTD scrapie isolates. Although the signal from white-tailed deer CWD cervical spinal cord remained strong, signals from WTD scrapie isolates were either greatly reduced or completely absent when probed with P4 (Figure 2, panel D).

**Figure 3:**
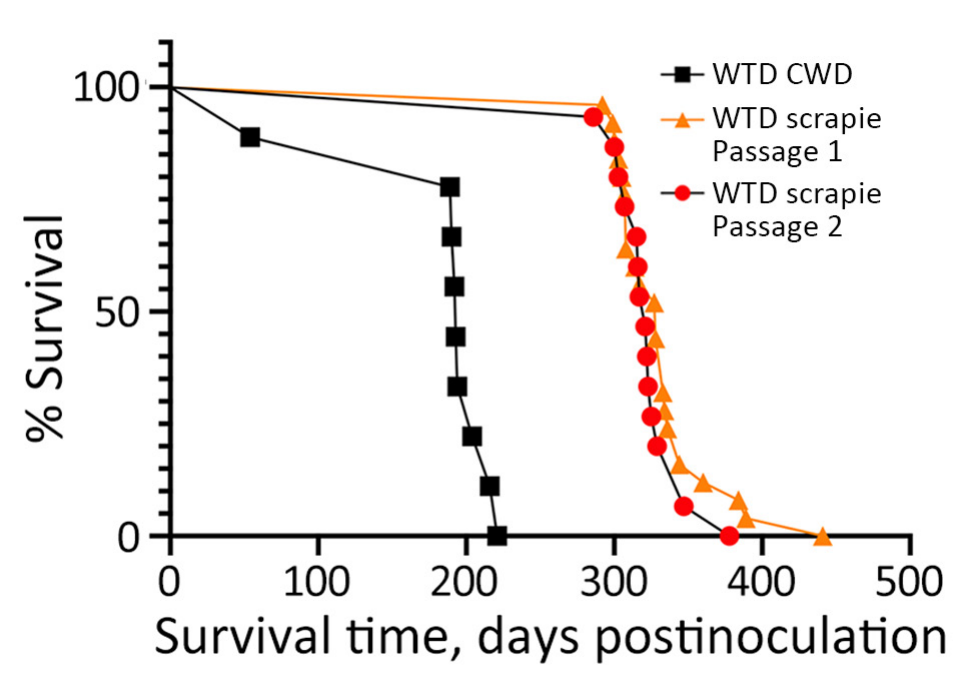
Survival curves for cervidized mice (Tg12) inoculated with brain material from white-tailed deer with CWD, passage 1 WTD scrapie agent, and passage 2 WTD scrapie agent in study of scrapie versus CWD in white-tailed deer. Incubation periods of mice inoculated with WTD scrapie agent were similar, whereas those inoculated with CWD were significantly shorter. CWD, chronic wasting disease; WTD, white-tailed deer.

**Figure 4:**
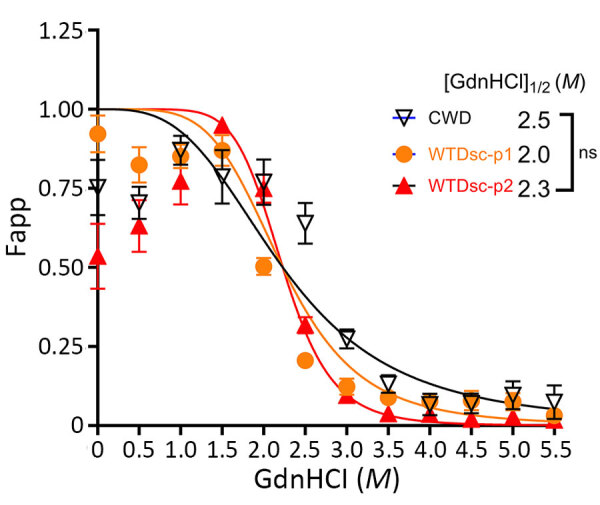
Denaturation curves comparing the conformational stability of scrapie (misfolded) form of prion protein (PrP^Sc^) in the cervical spinal cords of white-tailed deer with passage 2 WTD scrapie agent, passage 1 WTD scrapie agent, and CWD agent in study of scrapie versus CWD in white-tailed deer. The PrP^Sc^ conformational stability of like tissues did not differ significantly. CWD, chronic wasting disease; F*app*, apparent fractional change of unfolded PrP^Sc^; GdnHCl, guanidine hydrochloride; M, molar; WTDsc-p1, passage 1 WTD scrapie; WTDsc-p2, passage 2 WTD scrapie.

To identify potential strain differences, we inoculated cervidized mice (Tg12) with brainstem material (Table 1). We compared incubation periods of Tg12 mice inoculated with second-passage WTD scrapie agent (WTD scrapie P2; n = 15) with incubation periods of those inoculated with brainstem material from first-passage WTD scrapie agent (WTD scrapie P1; n = 25) and CWD agent. The incubation period in mice inoculated with brainstem from deer 1 (WTD scrapie P2) was similar to that in mice inoculated with WTD scrapie P1 (Figure 3). The average incubation time in mice inoculated with WTD scrapie P2 was 322 days after inoculation and did not differ significantly from that in mice inoculated with WTD scrapie P1 (340 days after inoculation). The incubation periods in mice inoculated with either WTD scrapie P1 or P2 differed significantly (p<0.0001) from those in mice inoculated with CWD agent, for which average incubation period was 199 days after inoculation (WTD CWD, n = 9). Attack rates for the 3 cohorts of mice were high (94%–100%). We performed conformational stability assays to determine if phenotypic differences between WTD scrapie passages and CWD were associated with differences in resistance of PrP^Sc^ to increasing concentrations of denaturant. After denaturation by guanidine hydrochloride, there was no difference in the conformational stability of the misfolded prion protein from the cervical spinal cords of white-tailed deer with second-passage WTD scrapie, first-passage WTD scrapie, or CWD (Figure 4). Therefore, molecular and mouse bioassay differences are not associated with differences in the conformational stability of PrP^Sc^.

## Discussion

**Figure 5:**
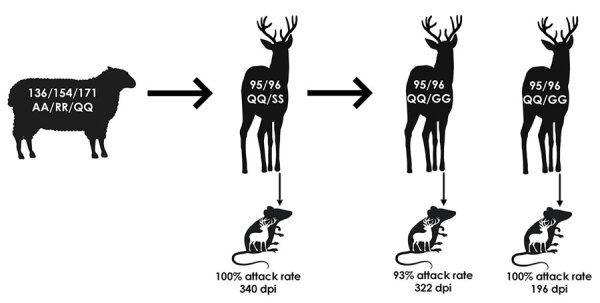
Summary of inoculum data, corresponding attack rates, and cervidized mice bioassays in study of scrapie versus chronic wasting disease in white-tailed deer. A, alanine; dpi, days postinoculation; G, glycine; Q, glutamine; R, arginine; S, serine.

Our study demonstrates that the WTD scrapie agent can be efficiently transmitted to wild-type white-tailed deer (Figure 5). After oronasal inoculation with the WTD scrapie agent, all 3 wild-type (QQ95/GG96) white-tailed deer displayed clinical signs and were positive for PrP^Sc^ in multiple nervous and lymphoreticular tissues (100% attack rate). Spongiform lesions, PrP^Sc^ accumulation, and molecular phenotypes of second-passage WTD scrapie were similar to those of the WTD scrapie inoculum. White-tailed deer are susceptible to infection with scrapie agents from various sources (*23*–*25*,*35*). Even when white-tailed deer at the lowest risk for CWD infection (SS96) (*33*,*36*,*37*) were exposed to classical sheep scrapie, they all succumbed to the disease (*23*–*25*). Unlike the initial passage of WTD scrapie agent in white-tailed deer (*23*), all brain tissues from our study exhibited a consistent molecular profile, probably because of the WTD scrapie agent stabilizing on the white-tailed deer PrP and differential neural prion selection (*38*). Further studies are needed to investigate the role that *PRNP* polymorphisms play in the disease progression of WTD scrapie in white-tailed deer (*8*,*39*,*40*).

WTD scrapie remains different from CWD on second passage in white-tailed deer. WTD scrapie differs from CWD in that WTD scrapie PrP^Sc^ accumulates in the retinal ganglion cells (Figure 1, panels E–G), has a shorter PK-resistant core (Figure 2, panels C, D), and has longer incubation periods in mice (Figure 3). Those differences did not result from differences in genotype (*41*), PrP^Sc^ conformational stability (Figure 4), or relative quantity of PrP^Sc^ in the inoculum (Table 1, 1% wt/vol) (*42*). Although many CWD strains in cervids have been characterized (*5*), none are able to address the longstanding hypothesis that classical sheep scrapie may be the origin of CWD in cervids (*43*). Our evidence suggests that WTD scrapie differs from CWD in white-tailed deer. Nevertheless, our evidence is limited to 2 experimental passages and the genotypes of deer used for those passages because genotype can affect prion transmission characteristics (*7*,*8*,*25*,*44*). Evaluating how the scrapie agent evolves in white-tailed deer requires subsequent passages in white-tailed deer of varying genotypes.

WTD scrapie has not been detected in wild or farmed cervids. If WTD scrapie were to be detected in cervids, management would remain consistent with current measures for CWD. WTD scrapie, like CWD, is lymphotropic. Lymphotropism occurs early in disease progression before neuroinvasion and indicates that the animal is shedding PrP^Sc^ into its environment and contaminating it (*26*–*28*). Although the WTD scrapie agent propagates effectively on white-tailed deer PrP, the only reported cases have been through experimental exposure. Because of the National Scrapie Eradication Program in 2001, cases of classical scrapie in farmed sheep have dramatically dropped and no case of classical scrapie has detected in the United States since January 2021 (https://www.aphis.usda.gov/sites/default/files/scrapie-quarterly-report-june-2024.pdf). The potential for zoonoses of cervid-derived PrP^Sc^ is still not well understood (*6*,*18*,*45*–*47*); however, interspecies transmission can increase host range and zoonotic potential (*48*–*50*). Therefore, to protect herds and the food supply, suspected cases of WTD scrapie should be handled the same as cases of CWD.
